# Negatively-Biased Credulity and the Cultural Evolution of Beliefs

**DOI:** 10.1371/journal.pone.0095167

**Published:** 2014-04-15

**Authors:** Daniel M. T. Fessler, Anne C. Pisor, Carlos David Navarrete

**Affiliations:** 1 Department of Anthropology and Center for Behavior, Evolution, and Culture, University of California, Los Angeles, Los Angeles, California, United States of America; 2 Department of Anthropology, University of California, Santa Barbara, Santa Barbara, California, United States of America; 3 Department of Psychology, Michigan State University, East Lansing, Michigan, United States of America; Durham University, United Kingdom

## Abstract

The functions of cultural beliefs are often opaque to those who hold them. Accordingly, to benefit from cultural evolution’s ability to solve complex adaptive problems, learners must be credulous. However, credulity entails costs, including susceptibility to exploitation, and effort wasted due to false beliefs. One determinant of the optimal level of credulity is the ratio between the costs of two types of errors: erroneous incredulity (failing to believe information that is true) and erroneous credulity (believing information that is false). This ratio can be expected to be asymmetric when information concerns hazards, as the costs of erroneous incredulity will, on average, exceed the costs of erroneous credulity; no equivalent asymmetry characterizes information concerning benefits. Natural selection can therefore be expected to have crafted learners’ minds so as to be more credulous toward information concerning hazards. This negatively-biased credulity extends general negativity bias, the adaptive tendency for negative events to be more salient than positive events. Together, these biases constitute attractors that should shape cultural evolution via the aggregated effects of learners’ differential retention and transmission of information. In two studies in the U.S., we demonstrate the existence of negatively-biased credulity, and show that it is most pronounced in those who believe the world to be dangerous, individuals who may constitute important nodes in cultural transmission networks. We then document the predicted imbalance in cultural content using a sample of urban legends collected from the Internet and a sample of supernatural beliefs obtained from ethnographies of a representative collection of the world’s cultures, showing that beliefs about hazards predominate in both.

## Introduction

Cultural evolution resembles biological evolution in some respects, and differs in others. As in biological evolution, the impact of information on the fitness of individuals and groups carrying it is a central determinant of the extent to which that information succeeds or fails in the arena of competing variants. However, the pathways for information transmission in cultural evolution are more diverse than in biological evolution [1], [2]. As a consequence, in addition to being driven by the fitness of information carriers, cultural evolution is also shaped by the extent to which a given variant is attractive to, retained by, and transmitted by human minds. The attractiveness, retainability, and transmissibility of a given cultural variant do not hinge solely on its utility, as they are also products of the extent to which the variant is congruent with features of learners’ minds. Patterns evident in a culture at a large scale thus in part reflect features common to the minds of those who hold the given culture, as such patterns are the result of the aggregated propensity of learners to acquire, retain, and transmit some beliefs and practices more than others [3]–[7].

Although a variety of features of learners’ minds have been explored in regard to their impact on cultural evolution, with only a few exceptions [8], [9], the attributes examined are not central to information acquisition and use, and hence their effects on cultural evolution are incidental (e.g., [10]–[12]). We propose that cultural evolution is importantly influenced by two linked features of learners’ minds–general negativity bias and its uniquely human extension, negatively-biased credulity–that play key roles in information acquisition and use. Here, we describe these features, present additional evidence of the existence of negatively-biased credulity, then demonstrate that, consistent with the expected effects of these biases writ large, beliefs about hazards predominate in domains in which information is exclusively social in origin.

Subjective reactions largely track fitness relevance, with fitness-reducing events typically being experienced as negative, i.e., eliciting aversive affective experiences and concomitant cognitions [13]. *Negativity bias* refers to the manner in which, compared to positive events, negative events more readily capture attention, are stored more readily in memory, are linked to a larger set of cognitions, and have greater motivational impetus [14], [15]. Negativity bias can be understood as reflecting an overarching pattern wherein the avoidance of imminent fitness decrements typically has a greater effect on fitness than does the pursuit of fitness enhancements, as, in general, the latter can be pursued only after the former have been addressed [14], [15].

Existing evidence suggests that negativity bias plays a role in the social transmission of information. News reports that induce fear are judged by viewers to be more important and relevant than those that do not [16]. Public opinion regarding economic outlooks is more strongly influenced by negative reports than by positive ones, even after controlling for the frequency of negative reporting [17]–[19], a pattern paralleled by the asymmetric effects of bad and good news regarding consumer sentiment on the stock market [20], [21]. At the affective level, eliciting negative emotions and related states should facilitate social transmission. Correspondingly, disgust, a negative emotion, figures prominently in past research: participants report greater likelihood of transmission for both non-social [22], [23] and social [24] information that elicits disgust, and they pursue information as a function of disgust content [22]. Disgust elicitation correspondingly predicts the distribution of urban legends [23], and is implicated in the longevity of etiquette rules [25]. More broadly, rumors reporting undesirable events spread more rapidly and more widely than those reporting desirable events, even when they are of equal importance and believability [26]. Arousal is a determinant of willingness to transmit information [27], and negative events generally elicit greater arousal than positive events [14], [15]. News reports that evoke high-arousal emotions are more likely to ‘go viral’ on the Internet, and anxiety is a principal driver in this regard [28]. Likewise, as both a state and a trait, anxiety is linked to the propensity to acquire and transmit rumors [29]–[34].

Evaluating pragmatic considerations such as impression management and informational utility, rumor researchers have also explored the positive contribution of credulity – how much the information is believed – to social transmission [31], [33], [35], [36]. Although not generally framed in these terms by students of rumor, the question of credulity can be seen as intimately linked to negativity bias. Contemplating the proximate cognitive mechanisms that contribute to credulity, Hilbig [37]–[39] proposed that negativity bias should extend to this realm, i.e., what he terms “negative information” should be more readily believed than “positive information”. It is important to underscore that most work on negativity bias concerns salience, memorability, and motivational impact – all factors that are logically distinct from credulity per se.

Building on previous basic research on negativity bias, investigations regarding communication about risks posed by technology indicate that people are indeed more likely to believe reports indicating that products are dangerous than they are to believe reports indicating that they are safe [40]–[43]. However, while noteworthy, such research does not reveal the extent to which these effects generalize beyond the topic of product safety. In multiple studies employing information concerning a broad range of topics, Hilbig [37]–[39] has demonstrated that, as predicted, negatively-framed information (much of which concerns the possibility of adverse outcomes) is believed at a higher rate than is positively-framed information. Follow-up studies reveal that this effect is not due to differences in the retrieval of prior knowledge, but instead likely stems from differences in processing fluency, and thus constitutes a true response bias [39]. Hilbig concludes by briefly noting that this bias may be functional, as fluency “often yields ecological validity” [39]. To the extent that Hilbig’s negatively- and positively-framed stimulus statements can respectively be construed as concerning hazards or opportunities, his suggestion of a functional bias articulates well with broader approaches that explain negativity bias as reflecting an evolutionary history characterized by the greater exigency of situations having the potential to decrease fitness relative to that of situations having the potential to enhance fitness [14], [15].

Consonant with the ubiquity of the asymmetrical fitness implications of hazards versus benefits, negativity bias occurs in many species [15]. Humans, however, diverge from other organisms in our reliance on culture, an attribute that has plausibly shaped the extension of negativity bias into the domain of social information transmission. Specifically, we argue that a consideration of the asymmetry in the costs attending credulity and incredulity across different categories of socially transmitted information provides an ultimate explanation for what we term *negatively-biased credulity,* an account that complements Hilbig’s proximate model of this phenomenon.

Humans are unique in both (a) their reliance on cultural information in addressing environmental and social adaptive challenges, and (b) the extent of their ability to acquire, use, improve, and transmit information from conspecifics, processes that, aggregated over time, generate a progressively larger corpus of useful cultural information. Importantly, if learners are to take advantage of the power of cultural evolution to solve fitness-relevant problems, they must be credulous. This is because not only is the utility of a belief or practice often not self-evident, but, moreover, it is frequently opaque to adherents, who often provide functionally extraneous rationales for their actions [44], [45]. However, credulity is accompanied by multiple costs. First, self-interested actors may deceive learners in order to exploit them [46]. Second, credulity increases the likelihood that non- or dys-functional beliefs and practices will be acquired, with subsequent declines in individual fitness [44].

The above considerations indicate that natural selection can be expected to have shaped the psychological mechanisms that play a role in culture acquisition so as to maximize the benefit/cost ratio of credulity [46]. An important factor in this equation will be the relative costs of two different types of errors, namely erroneous incredulity (failing to believe information that is true) and erroneous credulity (believing information that is false). Viewed in the larger context of issues of signal detection, these errors can be conceptualized, respectively, as false negatives and false positives. Whenever decision-making systems must act on the basis of imperfect information, a critical consideration is whether the relative costs of these two types of errors differ. By way of analogy, consider the design of household smoke detectors [47], [48]. It is prohibitively expensive to create smoke detectors that are perfectly accurate (i.e., devices that never sound an alarm in the absence of an actual fire, and always sound an alarm when a fire occurs). Smoke detectors should therefore be set to produce the less-costly error, namely false positives – we suffer the irritation of false alarms whenever we burn a piece of toast in order to enjoy the security of knowing that we will be alerted if a fire breaks out. The same considerations apply in the case of decision-making machinery crafted by natural selection, such that investigators should observe a consistent bias in the direction of whatever constitutes the less-costly error [47]–[55].

To understand how the above considerations apply to the question of credulity, consider two classes of cultural information, namely information concerning fitness-reducing hazards (e.g., which animals are dangerous, which plants are poisonous, which outgroups are hostile, etc.), and information concerning fitness-enhancing benefits (e.g., which animals are meaty, which plants are edible, which outgroups are friendly, etc.). With regard to cultural information concerning hazards, erroneous incredulity (i.e., a false-negative reaction) entails the costs suffered upon encountering the given hazard, while erroneous credulity (i.e., a false-positive reaction) entails only the costs of having taken unnecessary precautions. In the environments in which ancestral human populations evolved, ignoring accurate cultural information regarding hazards will often have led to serious injury or death, outcomes far more dire than the loss of time, energy, and opportunities resulting from having taken unnecessary precautions. Accordingly, in regard to cultural information concerning hazards, the costs of false negatives will have been larger on average than the costs of false positives. However, the situation is very different with regard to cultural information concerning benefits, as a false negative in this context (i.e., not believing cultural information that is, in fact, true) entails the costs of failing to exploit a useful opportunity, while a false positive (i.e., believing cultural information that is false) entails the costs of fruitlessly pursuing a spurious possibility. These respective costs will vary substantially from instance to instance; as a consequence, in contrast to the case of information concerning hazards, no overarching asymmetry is likely to have characterized this ratio in ancestral environments.

Given the greater costs of incredulity toward information concerning hazards relative to the costs of credulity toward such information, we should expect natural selection to have crafted a bias toward enhanced credulity. Because no equivalent asymmetry exists with regard to information concerning benefits, credulity in the latter domain should simply reflect the degree to which social learning is more advantageous than trial-and-error learning [45], [56]. Negatively-biased credulity can thus be understood as the output of a functional mechanism that enhances credulity toward socially transmitted information concerning hazards relative to the baseline level of credulity with which the individual approaches socially transmitted information concerning benefits.

### Overview of Studies

In order to provide an independent test for the existence of negatively-biased credulity, we conducted an initial investigation (Study 1) in which participants judged the likelihood that statements concerning hazards or benefits were true, predicting that the former should be believed more than the latter. Given that the utility of negatively-biased credulity is a function of both the prevalence of hazards and the extent to which the individual is able to cope with them, we posit that negatively-biased credulity may reflect the degree to which the individual views the world as dangerous. We tested this possibility in a second investigation (Study 2), combining the above methods with measures of individual differences. Having verified the existence of negatively-biased credulity and identified a key feature of individuals who exhibit it most strongly, we then moved from the individual level to the collective level, hypothesizing that, aggregated across individuals and information-transmission events, general negativity bias and negatively-biased credulity should together result in a predominance of beliefs concerning hazards in bodies of cultural information. We tested this prediction first in a set of urban legends circulating in the West (Study 3), then in a set of supernatural beliefs collected from a representative sample of the world’s cultures (Study 4).

### Ethics Statement

Studies 1 and 2 were examined and approved by the University of California, Los Angeles Institutional Review Board (Studies 3 and 4, which do not involve individual participants, were deemed exempt from review). Following the protocol approved by the Institutional Review Board, in both Study 1 and Study 2, participants were first presented with a web page describing the study procedures, any potential risks or discomforts, the identity and contact information of the first author, and the absence of compensation; participants then indicated their consent to participate by clicking a link to the study. The protocol approved by the Institutional Review Board dictated that consent be given anonymously.

### Data Archiving

Data for all studies described in this paper are archived at www.escholarship.org/uc/item/6v42v897.

## Study 1

Study 1 sought to test the negatively-biased credulity hypothesis using a broad sample of U.S. Internet users; the study thus provides a cross-cultural point of comparison for Hilbig’s [37]–[39] German Internet, university, and community samples. Moreover, rather than framing the information presented to participants in broadly positive or negative terms, we sought to overtly manipulate the extent to which this information addressed hazards or benefits. We predicted that participants would evince greater credulity toward information framed as potential hazards than toward information framed as potential benefits.

### Methods

#### Participants

Unpaid volunteers were recruited via advertisements, posted on Craigslist.org in major U.S. cities, for an online study titled “Truth or Trash? How Believable is the News Today?”. As we were relying on unpaid online volunteers, and thus were unsure as to the level of noise to expect in the data, a relatively large sample was recruited. Data were analyzed for 202 participants (129 females) ranging in age from 18 to 75 (*M = *37.29, *S.D.* = 14.02).

#### Materials and procedure

Unaware of Hilbig’s [37]–[39] research (see Introduction, above) at the time that we conducted our investigations, we independently converged on a method very similar to his (albeit with different contents), namely the use of differential framing to emphasize either the potential for losses from hazards or the potential for gains from beneficial opportunities. Kahneman and Tversky [57] pioneered this technique in their studies of loss aversion, the pattern wherein potential losses have greater motivational power than potential gains – a phenomenon that can itself be understood as a manifestation of general negativity bias [15]. Although Kahneman and Tversky were not concerned with issues of credulity, their technique is nevertheless valuable in the present context because it affords holding objective truth value constant across stimuli, thereby minimizing any effects of prior knowledge on judgments of believability (see also [39]).

Of particular applicability in the present context, the framing effects predicted to occur when logically equivalent statements are presented as concerning either hazards or benefits likely do not merely reflect limitations of human rationality [58]. Rather, we can understand these framing effects as due in part to the recipient’s assumption of communicative relevance [59], [60]. Statements that foreground hazards should reasonably be understood by the recipient as intentional warnings, while those that foreground benefits should be understood as intentional tips. Hence, while at first glance it may appear irrational to respond differently to the statements “X percent of people pursuing a benefit B via activity A suffer a cost C” and “(100-X) percent of people engaged in activity A obtain a benefit B without suffering a cost C,” the logic of discourse dictates that these are, in fact, very different statements – a speaker uttering the former is implicitly steering the listener clear of dangers that could befall her, while a speaker uttering the latter is implicitly encouraging the listener to exploit an opportunity that could benefit her. Thus, given our functionalist perspective on negatively-biased credulity as a mechanism to aid in the exploitation of socially transmitted information, statements akin to those employed by Kahneman and Tversky fall squarely within the proper domain of the postulated psychological system.

We first identified ten diverse facts that could be framed as either hazards or benefits. For each, we created two parallel statements, one emphasizing the hazard aspect, another emphasizing the benefit aspect. The statements were divided into two sets, one consisting of four statements regarding benefits and six regarding hazards, the other having the reverse ratio; these uneven ratios reduced cues as to the goals of the study. Only one of the two statements addressing a given topic appeared in a given set. To reduce attention to the details of the key statements, we then expanded both sets with six similarly worded distracter statements, subsequently excluded from analysis (see Appendix S1). Underscoring the communicative nature of these statements, we presented our study to participants as involving items excerpted from news media. Participants, randomly assigned to view one of the two sets, were asked to judge the likelihood that each statement was true using a 7-point scale (1 = Not at all True; 7 = Totally True). Item order was randomized and counterbalanced. The study ended with demographic items.

### Results

Participants’ net judgments of the believability of each message type (hazards versus benefits) were operationalized as the mean “true/not true” score for each domain. Participants were more likely to believe statements about hazards (*M* = 4.74; *S.D. = *0.85) relative to statements about benefits (*M* = 4.34, *S.D. = *0.81), a significant difference (*t*_201_ = 5.596, *p*<0.0001, *d = 0.55*). This result was robust to the exclusion of any one of the statements – no single statement was driving the significant difference between hazard credulity and benefit credulity.

## Study 2

Our agenda links the individual-level phenomena of general negativity bias and negatively-biased credulity with the group-level phenomenon of patterns in the cultural evolution of belief. The results of Study 1 provide independent support for the existence of negatively-biased credulity, a psychological mechanism that can be expected to operate as an attractor in cultural evolution [6], giving beliefs regarding hazards a competitive advantage in the marketplace of ideas. The social dynamics of information transmission constitute an intermediate level between individual psychology and cultural evolution, as it is in part via these dynamics that the former affects the latter. In turn, social dynamics are plausibly influenced by a number of features of individuals.

In assessing the differential impact of various individuals on social transmission, network researchers frequently consider social attributes, such as centrality in a network, frequency of contact with others, and so on [61]. However, in addition to social attributes, psychological features can plausibly also contribute to the degree to which individuals play differentiated roles in social transmission. We hypothesize that, while negatively-biased credulity is a species-typical trait, people will differ in the extent to which they evince this bias. Although here we limit our investigation to psychological differences, and do not explore transmission behavior, we suspect that such differences may influence the social dynamics of information transmission, as individuals characterized by high levels of negatively-biased credulity may act as important nodes in a network, accepting and broadcasting an above-average number of beliefs about hazards.

If negatively-biased credulity reflects the greater costs of erroneous incredulity relative to erroneous credulity regarding information about hazards, then the utility of negatively-biased credulity is in part conditional on the base rate of fitness-reducing hazards in the individual’s local environment: if the individual lives in a relatively safe environment, then much socially transmitted information regarding hazards will be false, shrinking the net cost of incredulity and enlarging the net cost of credulity; the converse will be true if the individual lives in a relatively dangerous environment. This raises the possibility that the postulated mechanism is subject to adjustment such that individuals will differ in negatively-biased credulity as a function of the level of danger in their environment.

The costs of encountering any given hazard depend in part on the personal and social resources that the individual brings to bear in coping with the hazard. Importantly, individuals differ in these attributes. As a consequence, the incredulity/credulity cost asymmetry will vary as a function of both the level of danger in the environment and the individual’s capacity for coping with that danger. Subjective perceptions of the level of danger in the world can plausibly be viewed as in part reflecting the combination of past encounters with hazards and self-assessed capabilities for addressing them [62]. Accordingly, if negatively-biased credulity is facultatively adjusted, then this trait should be positively correlated with the extent to which the individual perceives the world to be dangerous. Alternately, framed in proximate terms, whether as a result of experience, personality, or both, individuals who believe the world to be full of hazards will find novel statements concerning hazards to be more plausible than will individuals who expect hazards to be few and far between, as such statements will be consistent with the former’s expectations, and inconsistent with the latter’s. Importantly, the relationship between perceptions of the world as dangerous and the degree of negatively-biased credulity evinced should be independent of the extent to which the individual is credulous in general (i.e., outside of issues of hazards or benefits). At the ultimate level, this is because the utility of adjusting credulity as a function of the level of danger in the environment is limited to information regarding hazards. At the proximate level, expectations of danger should shape reactions to new information concerning hazards, but should be orthogonal to information concerning benefits, as the frequency of benefits is generally independent of the frequency of hazards. To test these predictions, we replicated Study 1, adding measures intended to gauge (a) individuals’ perception of their environment as dangerous, and (b) their general credulousness. As the goal of this study was to explore individual differences not examined in Study 1, we increased the target sample size five-fold (n = 1000) compared to the prior study, as we presumed that such a large increase would maximize the likelihood of capturing a substantial range of variation across participants.

### Methods

#### Participants

Recruitment and framing were identical to Study 1. Data were analyzed for 977 participants (578 females) who answered all questions presented; participants ranged in age from 18 to 81 (M = 39.06, S.D. 14.34).

#### Materials and procedure

In addition to the materials employed in Study 1, participants also responded to items taken from a measure designed to assess perceptions of danger in one’s environment [63], evaluated on a scale of 1 (Strongly Disagree) to 9 (Strongly Agree) (see Appendix S2). Four items, employing similar scales, then assessed both credulity in general, and credulity toward news sources in particular (see Appendix S2). Demographic items followed.

### Results and Discussion

Replicating the results of Study 1, participants were more credulous toward statements concerning hazards (M = 4.59, S.D. = 0.93) than toward statements concerning benefits (M = 4.16, S.D. = 0.88), *t*_976_ = 12.72, *p*<0.0001, *d = .58*), a difference again robust to the exclusion of any single statement.

To test the extent to which negatively-biased credulity is linked to subjective perceptions of the level of danger in the world, we conducted a multiple, multivariate regression analysis where hazard credulity, benefit credulity, and their difference score (M = .43, S.D. = 1.06) were the dependent variables, and subjective perception of danger (*M = *4.20, S.D. = 1.98) and general credulity (*M = *4.22, S.D. = 1.74) were the independent variables. The analysis revealed that subjective perception of danger was significantly linked to hazard credulity, *β = *.08, *t*_974_ = 2.48, *p = *.013, but not to benefit credulity, *t*_974_<1, while general credulity was linked to benefit credulity, *β = *.07, *t*_974_ = 2.48, *p = *.02, but not to hazard credulity, *t*_974_<1. As expected, subjective perception of danger was positively associated with the difference score measuring a bias of hazard credulity relative to benefit credulity, *β = *.08, *t*_974_ = 2.85, *p = *.004, but general credulity was not, *β = *.03. *t*_974_ = −1.54, *p = *.123. A cross-equation contrast confirmed that the hazard credulity/subjective perception of danger slope differed significantly from the benefit credulity/subjective perception of danger slope, *F*(1, 974) = 8.12, *p* = .005. Controlling for gender and age did not significantly affect these relationships.

In addition to replicating the results of Study 1 regarding the existence of negatively-biased credulity, consistent with our predictions, Study 2 revealed that the belief that the world is dangerous correlates with credulity toward statements concerning hazards, but not with credulity toward statements concerning benefits, a pattern that is independent of general credulousness. This suggests that individuals who believe the world to be dangerous may constitute important nodes in cultural transmission processes, as congruence between their priors and information concerning hazards may lead them to be particularly prone to differentially retain and propagate hazard information.

## Study 3

Both negatively-biased credulity and more general negativity bias operate at the level of individual minds. Iterated over many individuals and many transmission events, the differential propensity to acquire, hold, act on, and transmit different belief variants entailed by these biases should shape the content of culture. The conjunction of negatively-biased credulity and more general negativity bias thus predicts that, all else being equal, beliefs regarding hazards should be more common than beliefs regarding benefits. We can expect this effect to be most marked in those cultural domains that are removed from an objective basis, as, in such domains, it will be more difficult for forces in cultural evolution favoring accuracy to undercut the effects of the dual biases. Consonant with this supposition, observers have long speculated that folk beliefs appear to be dominated by harmful rather than helpful entities [64]. To test for the imbalance among folk beliefs of the frequency of hazard information relative to that of benefit information predicted by the bias hypothesis, we first examined urban legends circulating on the Internet.

### Methods

Drawing on prior work on this subject [65], [66], we define urban legends (henceforth ‘ULs’) as untrue accounts of events that a) ostensibly occurred in the present or recent past, in settings familiar to the audience, b) are intended to be both believable and believed, c) circulate widely in a social environment, and d) have a wide audience that does indeed believe them to be true or likely to be true. ULs were collected between July 15 and August 22, 2008 from the six principal web sites, as ranked by Google, devoted to the subject (see Appendix S3). ULs were selected on the basis of frequency of circulation, as indexed by the following criteria: a) categorized by one or more of the web sites as “most popular”; b) present on two or more of the six web sites (when duplicates occurred across web sites, the more detailed version was retained). Because political campaigns actively disseminate negative information about rivals, to avoid biasing the result in the predicted direction, ULs concerning political candidates were excluded.

Seven undergraduate students (five anthropology majors, and two biology majors) were recruited to serve as coders; all were naïve to both the specific hypothesis at issue and the general question of factors that might determine the frequency of different types of information in ULs. The coders evaluated a sample of 220 ULs, determining whether each UL described a hazard (defined as “something that imposes harm or other costs on those who encounter it”) and/or a benefit (“something that provides resources, opportunities, or other good things”). Each UL was coded in a binary fashion (Yes/No) on both items, with hazard and benefit category ratings being non-exclusive, producing 1,540 ratings for each question. Inter-rater reliability was validated in a two-way random effects model for absolute agreement, and yielded an intra-class correlation coefficient (ICC) for a between-rater mean of.77 across hazard ratings and.87 across benefits ratings. Coders also determined whether the UL addressed the physical environment (44.0% of ULs), animals (8.4%), supernatural forces (4.3%), and/or the social world (43.2%).

### Results

The benefit category contained 375 (out of a possible 1,540) “Yes” ratings, while the hazard category contained 1,198 (out of a possible 1,540) “Yes” ratings, indicating that hazard information appears approximately three times as frequently as benefit information. To statistically assess this ratio without inflating the degrees of freedom above the number of ULs, we first tallied the number of “Yes” benefit ratings and “Yes” hazard ratings for each UL, producing 220 tallies for each category ranging from 0 to 7 (0 = no judges gave the UL a “Yes” rating, 7 = all judges gave it a “Yes” rating). We then calculated the tally means within each content category and compared the two. A two-group mean comparison test revealed a significantly higher “Yes” count for hazards (M = 5.40, S.D. = 1.89) compared to that found for benefits (M = 1.70, S.D. = 2.27), *t*_219_ = 14.62. *p*<1×10^−33^. These findings are consistent with the notion that, when scaled up over multiple actors, generalized negativity bias and negatively-biased credulity produce an imbalance in socially transmitted cultural content.

## Study 4

If generalized negativity bias and negatively-biased credulity are panhuman features of mind, then the imbalance in cultural content found in our sample of urban legends in the English-speaking West should also occur in beliefs sampled across diverse cultures. By virtue of their frequency around the world, supernatural beliefs afford testing this prediction; additionally, only 4.3% of the urban legends examined in Study 3 concerned supernatural forces, enhancing the independence of such a test. We therefore examined the frequency of hazard information in a representative sample of the world’s supernatural beliefs.

### Methods

Assistants, naïve to the hypothesis, collected supernatural beliefs from the 60 cultures described in the Probability Sample Files of the Human Relations Area Files (HRAF), a representative sample of the world’s cultures [67]. Supernatural beliefs were identified using HRAF search codes for “Spirits and Gods,” “Religious Beliefs,” “Eschatology,” and “Avoidance and Taboos”. To preclude overweighting cultures having elaborate belief systems, only the first five beliefs encountered in each Sample File were collected (although as few as two per culture were found in some cases). The aforementioned HRAF search codes, which vary, in descending order, from broad to narrow, are listed in the order in which they were applied for each culture: to maximize the breadth of beliefs captured, assistants only moved down the list of codes if, for a given culture, the preceding code(s) had not yet yielded five beliefs. The vast majority of the beliefs collected thus derived from the categories “Spirits and Gods” and “Religious Beliefs”. Six of the coders employed in Study 3 were then recruited to evaluate the resulting sample of 219 supernatural beliefs using the same criteria applied in Study 3. These coders were again naïve to both the specific hypothesis at issue and the larger question of factors that might determine the frequency of different types of information in supernatural beliefs. Inter-rater reliability was validated as in Study 3, with an ICC mean of.86 for hazard ratings and.82 for benefit ratings.

### Results

The benefit category contained 556 (out of a possible 1,314) “Yes” ratings, while the hazard category contained 817 (out of a possible 1,314), indicating that hazard information appears approximately 1.5 times as frequently as benefit information. We assessed the significance of this difference using the method employed in Study 3, returning 219 tallies for each category ranging from 0 to 6 (0 = no judges gave a “Yes” rating, 6 = all judges gave a “Yes” rating), and then averaging them within category. A two-group mean comparison test revealed a significantly higher “Yes” count for hazards (*M = *3.73, *S.D.* = 2.24) compared to that found for benefits (*M* = 2.54, *S.D.* = 2.14), *t*_218_ = 4.75, *p*<0.0001. Hence, as predicted, information concerning hazards is substantially more common than information concerning benefits in supernatural beliefs.

## General Discussion

Replicating and extending Hilbig’s [37]–[39] findings from German participants, in two samples of U.S. adults, we demonstrated that people are more credulous of information regarding hazards than of information concerning benefits. Expanding existing functionalist perspectives on negativity bias into the realm of the acquisition and use of socially transmitted information, we view this negatively-biased credulity as reflecting a pattern wherein, in the environments in which humans evolved, the costs of erroneous incredulity toward information regarding hazards would, on average, have exceeded the costs of erroneous credulity. No equivalent asymmetry would have characterized the treatment of information regarding benefits, hence natural selection can be expected to have favored negatively-biased credulity. Our thesis is agnostic as to whether such asymmetry reflects, on the one hand, a novel manifestation in humans of the same processes responsible for negativity bias in other species, or, on the other hand, a serially homologous derived trait [68], [69] operating in parallel with such processes: for the present purposes, what matters is simply that people evince greater credulity toward information concerning hazards.

Our framework introduces the possibility that negatively-biased credulity may be facultatively adjusted, as its utility depends in part on the actual risks posed by hazards in the individual’s environment. Subjective perceptions of the extent to which the world is dangerous capture a combination of one’s past encounters with hazards and an assessment of one’s ability to effectively address hazards. Consonant with the above, we demonstrated that credulity toward hazard information, but not toward benefit information, is correlated with perceptions that the world is dangerous. Although we interpret this relationship in terms of the effects of self-perceived vulnerability to hazards on credulity, because the data are correlational, we cannot rule out reverse causality, i.e., some people may perceive the world as more dangerous because, due to some additional factor, they are more credulous toward information about hazards.

Moving from the level of individual psychology to the level of information shared by members of a society, the combined effects of general negativity bias and negatively-biased credulity in the minds of learners should constitute an attractor [6] that shapes the contours of cultural evolution: culture can be expected to exhibit an imbalance wherein information regarding hazards is more prevalent than information regarding benefits. This imbalance should be particularly pronounced in domains in which the information at issue lacks, or is distant from, an objective basis, as this reduces the influence of selection pressures in cultural evolution that favor functional utility – and thus, frequently, accuracy – in cultural information. We found the predicted imbalance in two widely disparate samples, one a set of urban legends circulating on the Internet in the contemporary English-speaking West, the other a collection of supernatural beliefs extracted from ethnographic descriptions of a representative sample of the world’s cultures.

Being the product of innumerable instances of information acquisition, retention, and transmission, urban legends and supernatural beliefs are sufficiently removed from the individual minds that shaped them that we cannot say for certain what the relative contributions of general negativity bias and negatively-biased credulity are to the resulting cultural content. Nevertheless, our findings provide the basis for further investigations aimed at illuminating the manner in which features of learners’ minds influence the shape of culture. Notably, social dynamics operate at an intermediate level in this process. Regardless of the direction of causality underlying the correlation, our observation that people who view the world as dangerous exhibit enhanced negatively-biased credulity suggests that such individuals may be critical nodes in the transmission chains that mold culture: being more likely to acquire, retain, and transmit beliefs concerning hazards than are others in their population, those who see the world as dangerous may exercise an outsized influence on the eventual imbalance in the contents of culture between information concerning hazards and information concerning benefits. Future investigations aimed at capturing the processes of social information transmission and the structure of networks involved therein may therefore be strengthened by examining individual variation along this dimension.

Our thesis that general negativity bias and negatively-biased credulity are universal features of the human mind predicts that the resulting imbalance in beliefs should be common across cultures. Beyond this overarching pattern, cultures may differ substantially in the extent of this imbalance. In part this is because, for any given society, the asymmetry in the costs of errors with regard to credulity concerning hazard information will be a function of the base rate of actual hazards in the given environment. The costs of failing to believe true statements about hazards hinge on the likelihood that the given hazard will be encountered. This probability will be higher in societies located in relatively dangerous environments (e.g., many natural hazards; high rates of violence; etc.) than in societies located in relatively safe environments. As a consequence, in dangerous environments, the cost/benefit ratio is shifted even further toward greater credulity regarding hazard information. If, as we have suggested, such credulity is shaped in part by subjective perceptions of vulnerability, then it is plausible that negatively-biased credulity will partially track the base rate of actual hazards in the environment. In turn, greater credulity in objectively more dangerous environments will enhance the opportunity for spurious beliefs about hazards to proliferate in the given culture, leading to the prediction that both negatively-biased credulity and the prevalence of beliefs about hazards will be greater in dangerous environments than in safe environments, and, moreover, that the latter difference will persist even after controlling for differences in objective danger. Furthermore, if subjective vulnerability is itself shaped in part by cultural information (i.e., individuals feel more vulnerable when the cultural context depicts many hazards than when it depicts few), then, by virtue of the impact of subjective vulnerability on the success or failure of beliefs in the marketplace of ideas, feedback loops may occur wherein both prevailing levels of subjective vulnerability and the prevalence of beliefs about hazards change over time without corresponding alterations in the objective base rate of hazards in the environment. Due to the core asymmetry in costs underlying negatively-biased credulity, we can expect such runaway processes to be similarly biased: it should be easier to increase than to decrease subjective vulnerability, and, correspondingly, positive feedback processes that successively increase the frequency of hazard beliefs relative to the objective prevalence of hazards should be more common than negative feedback processes that generate the reverse pattern. Indeed, observing the extreme degree to which Melanesian cultures – which occupy objectively dangerous environments – populate cosmologies with dangerous forces and entities, Schwartz [70] posited the existence of self-reinforcing ‘paranoid ethoses’.

As noted in the Introduction, credulity is a prerequisite for the acquisition and exploitation of valuable knowledge produced by cumulative cultural evolution in large part because the functional utility of such information is often opaque to the learner, and, moreover, frequently to its expert adherents as well. Such opacity has been cited as the adaptive consideration driving overimitation, the pattern, largely unique to humans, wherein, once they have grasped the apparent goal of a given model’s actions (which need not be isomorphic with its true utility), learners imitate even seemingly extraneous features of the model’s behavior [71]. As such, overimitation can plausibly be construed as a form of credulity operating in contexts in which information transmission is mediated primarily by behavior rather than language. If so, then we should expect negatively-biased credulity to be evident here as well, generating the prediction that overimitation will be more pronounced when the actions of the model address hazards than when said actions address benefits. In turn, at the level of cultural evolution, this pattern may contribute to the development of rituals, as rituals both frequently address hazards and are characterized by stereotyped behaviors that are transmitted with a high degree of fidelity [72], [73].

The cultural evolution of supernatural beliefs is likely shaped in part by the positive effects on cooperation of beliefs in moralistic supernatural entities [74], [75]. Although we did not differentiate between punitive moralistic entities and other types of hazards in our ethnographic sample, such an effect cannot explain our overall results, as only a small fraction of the urban legends that we examined concerned supernatural forces. The success of modern Christianity might lead observers to presume that supernatural beliefs are generally a source of comfort to their holders. Granted, the predominance of hazard information in our cross-cultural corpus of beliefs may not translate into anxiety in believers since, for example, beliefs might offer sense-making accounts of why hazards exist. However, given the demonstrated role of anxiety in enhancing cultural transmission, the success of hazard information in the marketplace of ideas likely reflects its capacity to create, not alleviate, aversive subjective experiences. Rather than reflecting the impact of psychological attractors, the success of proselytizing religions that depict beneficent but moralistic deities may therefore primarily reveal larger-scale dynamics wherein supernatural beliefs that further within-group cooperation and altruism are favored by cultural group selection [76]–[79], thus illustrating the complex, multi-level nature of cultural evolution.

## Supporting Information

Appendix S1 **Materials Used in Studies 1 and 2 to Evaluate Hazard Credulity Bias.**
(DOCX)

Appendix S2 **Additional Measures Employed in Study 2.**
(DOCX)

Appendix S3 **Sources of Urban Legends Employed in Study 3.**
(DOCX)
