# Supplementary material for: Negatively-Biased Credulity and the Cultural Evolution of Beliefs
Source: PLoS One. 2014 Apr 15;9(4):e95167. doi: 10.1371/journal.pone.0095167 (PMC3988160; doi:10.1371/journal.pone.0095167)
Supplement: Appendix S1 — Materials Used in Studies 1 and 2 to Evaluate Hazard Credulity Bias. (DOCX) [file pone.0095167.s001.docx]

**Supporting Information to Accompany**

**Fessler, Pisor, & Navarrete’s**

***Negatively-Biased Credulity and the Cultural Evolution of Beliefs***

**Appendix S1: Materials Used in Studies 1 and 2 to Evaluate Hazard Credulity Bias**

*Highlighted items are dependent variables; items without highlights are similarly structured distractors.*

**SET 1**

For each of the statements that follow, please indicate, by checking the appropriate box, how confident you are that the statement is **TRUE** or **FALSE**.

**1.** Although proponents consider German shepherds loyal and intelligent pets, a recent study in the U.S. notes that this breed is responsible for 11% of dog attacks.

1 2 3 4 5 6 7

I’m absolutely I’m absolutely

certain this certain this

statement is **FALSE** statement is **TRUE**

**2.** On average, four years after opening, 44% of small businesses are turning a profit and still in business.

1 2 3 4 5 6 7

I’m absolutely I’m absolutely

certain this certain this

statement is **FALSE** statement is **TRUE**

**3.** Many people find rare ground beef patties delicious. However, 13% of ground beef patties contain bacteria that could cause illness if eaten rare.

1 2 3 4 5 6 7

I’m absolutely I’m absolutely

certain this certain this

statement is **FALSE** statement is **TRUE**

**4.** Homes that border a wilderness preserve are typically 11% more valuable than homes in the same neighborhood that do not border the wilderness.

1 2 3 4 5 6 7

I’m absolutely I’m absolutely

certain this certain this

statement is **FALSE** statement is **TRUE**

**5.** In the case of suspected cardiac arrest, an automatic external defibrillator (AED) can recognize heart arrhythmias and apply an electric shock if needed, thus saving lives. If treated early with an AED, over 50% of people undergoing cardiac arrest will survive.

1 2 3 4 5 6 7

I’m absolutely I’m absolutely

certain this certain this

statement is **FALSE** statement is **TRUE**

**6.** When civil litigation cases go to trial, 60% of plaintiffs lose, winning no money, and often having to pay attorney fees.

1 2 3 4 5 6 7

I’m absolutely I’m absolutely

certain this certain this

statement is **FALSE** statement is **TRUE**

**7.** In heavy city traffic, bicyclists typically arrive at their destination twice as fast as motorists traveling the same distance by car.

1 2 3 4 5 6 7

I’m absolutely I’m absolutely

certain this certain this

statement is **FALSE** statement is **TRUE**

**8.** LASIK eye surgery leads to lasting complications -- including double vision -- in 5% of patients who undergo the procedure.

1 2 3 4 5 6 7

I’m absolutely I’m absolutely

certain this certain this

statement is **FALSE** statement is **TRUE**

**9.** Owning a dog has many benefits: People who own dogs are 55% more likely to get the amount of exercise recommended for good health than people who don’t own dogs.

1 2 3 4 5 6 7

I’m absolutely I’m absolutely

certain this certain this

statement is **FALSE** statement is **TRUE**

**10.** Becoming a professor is a less desirable career path than you might think, as few tenure-track positions are available: only 34% of the faculty positions offered at US colleges and universities are tenure-track.

1 2 3 4 5 6 7

I’m absolutely I’m absolutely

certain this certain this

statement is **FALSE** statement is **TRUE**

**11.** Chemotherapy is known to slow or stop tumor growth in many cancer patients. However, at least 30% of young people and at least 60% of people over 40 who undergo chemotherapy are subsequently infertile.

1 2 3 4 5 6 7

I’m absolutely I’m absolutely

certain this certain this

statement is **FALSE** statement is **TRUE**

**12.** Many people enjoy jogging. Adults who run regularly have a significantly lower risk of heart disease than those who do not run regularly.

1 2 3 4 5 6 7

I’m absolutely I’m absolutely

certain this certain this

statement is **FALSE** statement is **TRUE**

**13.** Gardasil has been shown to protect women from types of human papilloma virus that cause the majority of cervical cancers. 93% of women who receive the vaccine experience no serious side effects.

1 2 3 4 5 6 7

I’m absolutely I’m absolutely

certain this certain this

statement is **FALSE** statement is **TRUE**

**14.** Short-term momentum investors, who buy when high performance stocks are on sale and sell when the market price of their stocks increases, overestimate the strength of their strategy: critics argue that, due to changes in the market, the momentum strategy can be used effectively only 15 to 20% of the time.

1 2 3 4 5 6 7

I’m absolutely I’m absolutely

certain this certain this

statement is **FALSE** statement is **TRUE**

**15.** 99% of people are not allergic to peanuts and can benefit from their high fiber content.

1 2 3 4 5 6 7

I’m absolutely I’m absolutely

certain this certain this

statement is **FALSE** statement is **TRUE**

**16.** Many people find crab fishing alluring as a quick job that pays well; however, fishing is a dangerous job, taking over 100 lives annually per 100,000 fishermen.

1 2 3 4 5 6 7

I’m absolutely I’m absolutely

certain this certain this

statement is **FALSE** statement is **TRUE**

**SET 2**

For each of the statements that follow, please indicate, by checking the appropriate box, how confident you are that the statement is **TRUE** or **FALSE**.

**1.** Despite their fierce appearance, German shepherds are considered loyal and intelligent pets. A recent study in the U.S. notes that other breeds of dog are responsible for 89% of dog attacks.

1 2 3 4 5 6 7

I’m absolutely I’m absolutely

certain this certain this

statement is **FALSE** statement is **TRUE**

**2.** On average, four years after opening, 66% of small businesses have failed and gone out of business.

1 2 3 4 5 6 7

I’m absolutely I’m absolutely

certain this certain this

statement is **FALSE** statement is **TRUE**

**3.** Many people find rare ground beef patties delicious. 87% of ground beef patties are free of bacteria that could cause illness if eaten rare.

1 2 3 4 5 6 7

I’m absolutely I’m absolutely

certain this certain this

statement is **FALSE** statement is **TRUE**

**4.** Homes that border a wilderness preserve become 10% less valuable during the five years following a local wildfire than homes in the same neighborhood that do not border the wilderness.

1 2 3 4 5 6 7

I’m absolutely I’m absolutely

certain this certain this

statement is **FALSE** statement is **TRUE**

**5.** In the case of suspected cardiac arrest, an automatic external defibrillator (AED) can recognize heart arrhythmias and apply an electric shock if needed, thus saving lives. If used improperly, however, a layperson operating an AED can also receive a shock, injuring them.

1 2 3 4 5 6 7

I’m absolutely I’m absolutely

certain this certain this

statement is **FALSE** statement is **TRUE**

**6.** When civil litigation cases go to trial, 40% of plaintiffs succeed and win money.

1 2 3 4 5 6 7

I’m absolutely I’m absolutely

certain this certain this

statement is **FALSE** statement is **TRUE**

**7.** In heavy city traffic, bicyclists are typically twice as likely to be injured in a crash as motorists traveling the same distance by car.

1 2 3 4 5 6 7

I’m absolutely I’m absolutely

certain this certain this

statement is **FALSE** statement is **TRUE**

**8.** LASIK eye surgery improves vision to 20/20 in 90% of patients who undergo the procedure.

1 2 3 4 5 6 7

I’m absolutely I’m absolutely

certain this certain this

statement is **FALSE** statement is **TRUE**

**9.** Owning a dog has many costs: The average lawsuit over a dog attack costs the dog owner approximately $24,000.

1 2 3 4 5 6 7

I’m absolutely I’m absolutely

certain this certain this

statement is **FALSE** statement is **TRUE**

**10.** Becoming a professor is a more desirable career path than you might think, as tenure-track positions pay well: on average, tenure-track professors earn $81,000 a year.

1 2 3 4 5 6 7

I’m absolutely I’m absolutely

certain this certain this

statement is **FALSE** statement is **TRUE**

**11.** Chemotherapy is known to slow or stop tumor growth in many cancer patients. Up to 70% of people who undergo chemotherapy experience slowing or stopping of tumor growth.

1 2 3 4 5 6 7

I’m absolutely I’m absolutely

certain this certain this

statement is **FALSE** statement is **TRUE**

**12.** Although many people enjoy jogging, adults who run regularly are four times more likely to have a knee injury than the general population.

1 2 3 4 5 6 7

I’m absolutely I’m absolutely

certain this certain this

statement is **FALSE** statement is **TRUE**

**13.** Gardasil has been shown to protect women from types of human papilloma virus that cause the majority of cervical cancers. However, 7% of women who receive the vaccine experience serious side effects.

1 2 3 4 5 6 7

I’m absolutely I’m absolutely

certain this certain this

statement is **FALSE** statement is **TRUE**

**14.** Short-term momentum investors, who buy when high performance stocks are on sale and sell when the market price of their stocks increases, praise the strength of their strategy: proponents claim that the momentum strategy allows investors to profit from 70% of their stock purchases.

1 2 3 4 5 6 7

I’m absolutely I’m absolutely

certain this certain this

statement is **FALSE** statement is **TRUE**

**15.** 1% of people are allergic to peanuts and must not eat them despite their beneficial fiber content.

1 2 3 4 5 6 7

I’m absolutely I’m absolutely

certain this certain this

statement is **FALSE** statement is **TRUE**

**16.** Many people find crab fishing alluring as a quick job that pays well: fishermen often make $60,000 for three months work.

1 2 3 4 5 6 7

I’m absolutely I’m absolutely

certain this certain this

statement is **FALSE** statement is **TRUE**
