# Supplementary material for: Negatively-Biased Credulity and the Cultural Evolution of Beliefs
Source: PLoS One. 2014 Apr 15;9(4):e95167. doi: 10.1371/journal.pone.0095167 (PMC3988160; doi:10.1371/journal.pone.0095167)
Supplement: Appendix S2 — Additional Measures Employed in Study 2. (DOCX) [file pone.0095167.s002.docx]

**Supporting Information to Accompany**

**Fessler, Pisor, & Navarrete’s**

***Negatively-Biased Credulity and the Cultural Evolution of Beliefs***

**Appendix S2: Additional Measures Employed in Study 2**

**Beliefs in the Dangerousness of the World scale (Navarrete, 2005)**

1. I often fear for my safety

1 2 3 4 5 6 7 8 9

**Very Strongly Neutral Very Strongly**

**Disagree Agree**

1. I often fear for the safety of my family and/or friends.

1 2 3 4 5 6 7 8 9

**Very Strongly Neutral Very Strongly**

**Disagree Agree**

1. The world is a dangerous place.

1 2 3 4 5 6 7 8 9

**Very Strongly Neutral Very Strongly**

**Disagree Agree**

**Generalized Credulity scale (created by the authors)**

1. I am a skeptical person – unless I have seen it with my own eyes, I often do not believe what people tell me.

1 2 3 4 5 6 7 8 9

**Very Strongly Neutral Very Strongly**

**Disagree Agree**

1. Newspapers report objective facts – the things you read in the newspaper are true.

1 2 3 4 5 6 7 8 9

**Very Strongly Neutral Very Strongly**

**Disagree Agree**

1. I tend to believe what other people tell me – compared to most people I know, I am a very trusting person.

1 2 3 4 5 6 7 8 9

**Very Strongly Neutral Very Strongly**

**Disagree Agree**

1. I don’t believe anything I hear on television news programs – those guys just spread rumors.

1 2 3 4 5 6 7 8 9

**Very Strongly Neutral Very Strongly**

**Disagree Agree**
