# Supplementary material for: Negatively-Biased Credulity and the Cultural Evolution of Beliefs
Source: PLoS One. 2014 Apr 15;9(4):e95167. doi: 10.1371/journal.pone.0095167 (PMC3988160; doi:10.1371/journal.pone.0095167)
Supplement: Appendix S3 — Sources of Urban Legends Employed in Study 3. (DOCX) [file pone.0095167.s003.docx]

**Supporting Information to Accompany**

**Fessler, Pisor, & Navarrete’s**

***Negatively-Biased Credulity and the Cultural Evolution of Beliefs***

**Appendix S3: Sources of Urban Legends Employed in Study 3**

Material collected between July 15 and August 22, 2008

www.snopes.com

www.truthorfiction.com

www.geocities.com/rayman_7575/urbanlegendcentral.html

www.netscrap.com/netscrap.cfm?cat_show=Urban%20Folklore

http://urbanlegendsonline.com/classics.html

http://urbanlegends.about.com/od/reference/a/top_25_uls.htm
